# Uncertainties around net‐zero climate targets have major impact on greenhouse gas emissions projections

**DOI:** 10.1111/nyas.15285

**Published:** 2025-01-12

**Authors:** Michel G. J. den Elzen, Ioannis Dafnomilis, Leonardo Nascimento, Arthur Beusen, Nicklas Forsell, Joost Gubbels, Mathijs Harmsen, Elena Hooijschuur, Zuelclady Araujo Gutiérrez, Takeshi Kuramochi

**Affiliations:** ^1^ PBL Netherlands Environmental Assessment Agency The Hague The Netherlands; ^2^ Institute for Environmental Studies (IVM) Vrije Universiteit Amsterdam Amsterdam The Netherlands; ^3^ NewClimate Institute Cologne Germany; ^4^ Environmental Systems Analysis Group Wageningen University Wageningen The Netherlands; ^5^ Department of Earth Sciences‐Geochemistry Utrecht University Utrecht The Netherlands; ^6^ International Institute for Applied Systems Analysis (IIASA) Laxenburg Austria; ^7^ Copernicus Institute of Sustainable Development Utrecht University Utrecht The Netherlands

**Keywords:** climate change mitigation, climate policy, integrated assessment models, net‐zero targets, Paris Agreement, uncertainty

## Abstract

Globally, more than 100 countries have adopted net‐zero targets. Most studies agree on how this increases the chance of keeping end‐of‐century global warming below 2°C. However, they typically make assumptions about net‐zero targets that do not capture uncertainties related to gas coverage, sector coverage, sinks, and removals. This study aims to analyze the impact of many uncertainty factors on the projected greenhouse gas (GHG) emissions by 2050 for major emitting countries following their net‐zero pathways, and their aggregate impact on global GHG emissions. Global emission projections range from 23 to 40 gigatons of CO_2_ equivalent (GtCO_2_eq), with a median of 31 GtCO_2_eq. Our full range corresponds to about 40–75% of 2015 emission levels, which is much wider than the range of 30–45% reported by various integrated assessment models. The main factors contributing to this divergence are the uncertainty in the gas coverage of net‐zero targets and uncertainty in the socioeconomic baseline. Countries with net‐zero GHG targets by 2050 have a small range of 2050 emissions, while countries with net‐zero targets beyond 2050 and unclear coverage, such as China, India, and Indonesia, have a large range of emissions by 2050.

## Graphical abstract

Over 100 countries have adopted net‐zero targets to limit global warming. However, uncertainties in gas coverage, sinks, and socio‐economic baselines impact emissions projections. This study examines these uncertainties for major emitters, showing that 2050 global emissions could range from 23–40 GtCO_2_eq, wider than previous estimates. Countries with net‐zero by 2050 show smaller variability, while countries like China and India, with later or unclear net‐zero targets, show broader emission ranges.

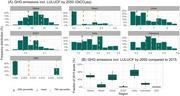

## INTRODUCTION

As of January 2024, more than 100 countries, covering approximately 81% of global greenhouse gas (GHG) emissions, have announced and/or adopted net‐zero targets.^1^ In many cases, these targets contain many uncertainties and ambiguities, for example, regarding what GHGs and sectors they cover and their net‐zero target year.^2^, ^3^ The pathway trajectories toward these net‐zero targets and how socioeconomic assumptions and near‐term climate policies influence them are additional sources of uncertainty.

In addition, there are uncertainties about the negative emissions from nature‐based carbon dioxide removal (CDR), such as carbon sequestration in forests and soils and CDR technologies, that is, biomass energy with carbon capture and storage and direct air carbon capture and storage.^4^, ^5^ The extent of deployment of both categories of CDR at the country level, as reported in the national long‐term plans and documents, has been analyzed in several studies.^4^, ^6^ Based on the assessment of these documents,^5^, ^7^ many countries plan to expand land‐based removals. However, none have yet committed to substantively scaling novel CDR methods, and many national documents lack transparency. CDR has also been extensively analyzed on a sectoral level in integrated assessment models.^8^, ^9^, ^10^

Together, these variations allow for different interpretations and achievements of the net‐zero targets, influencing the projected GHG emission pathways toward net‐zero. The global emissions pathways of the most recent net‐zero targets have already been analyzed in several studies,^2^, ^11^, ^12^, ^13^, ^14^, ^15^, ^16^, ^17^, ^18^ which make specific assumptions about the countries’ net‐zero targets regarding gas coverage, sinks, and removals. A systematic, peer‐reviewed analysis of how these uncertainties affect the projected emission for the latest net‐zero targets is still lacking. Earlier studies^19^, ^20^ have analyzed the impact of uncertainties on 2030 emissions projections implied by countries’ National Determined Contributions (NDCs) based on several uncertainty factors related to NDCs.

Against this backdrop, this study aims to analyze and quantify the impact of key uncertainty factors related to the net‐zero targets (cut‐off date of January 30, 2024) on global and national emission pathways toward these targets, as well as on abatement costs and carbon prices, and global temperature increase. We present detailed results for both the global level and for the six largest emitting economies (Brazil, China, EU27, India, Indonesia, and the United States), which are collectively responsible for 65% of global GHG emissions. We also analyze the individual contributions of specific uncertainty factors to this overall uncertainty.

**FIGURE 1:**
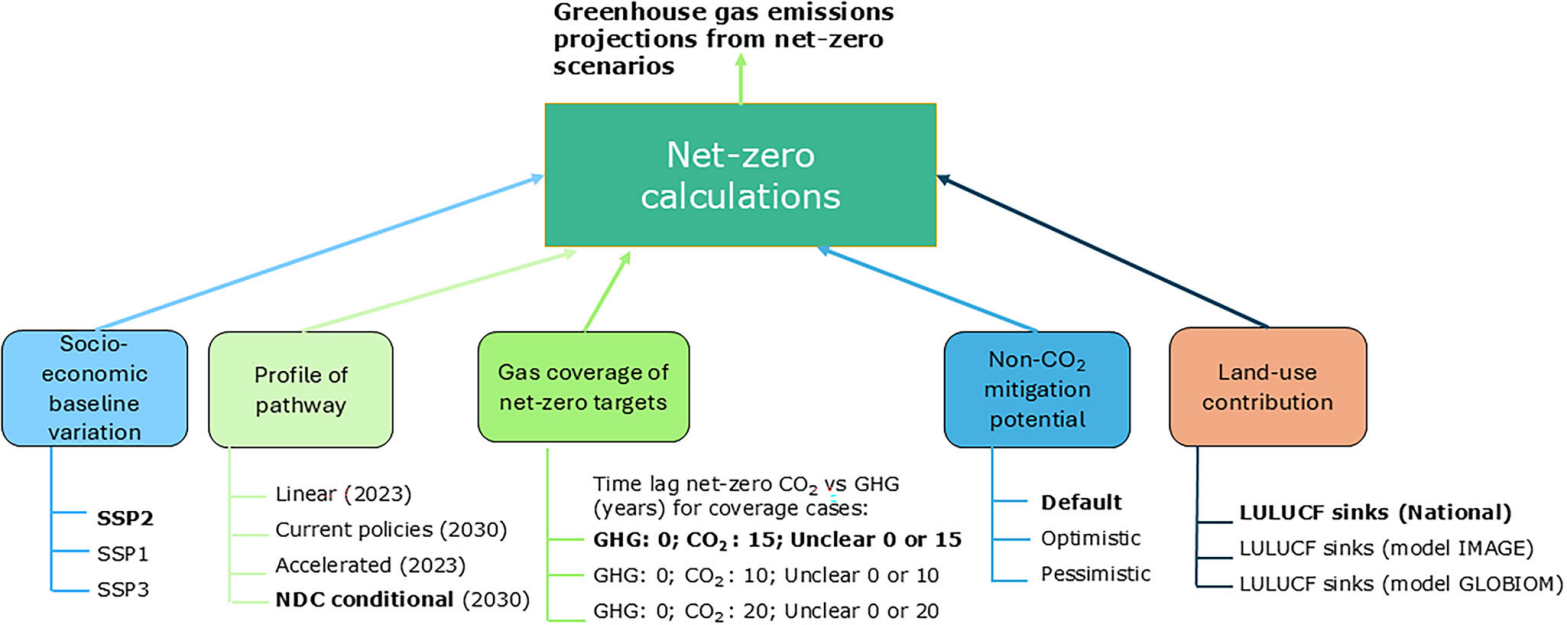
Overview of the uncertainty factors analyzed, including the different subcategories of each factor. The bold text represents the default setting. Abbreviations: GHG, greenhouse gas; LULUCF, land‐use, land‐use change and forestry; NDC, National Determined Contributions; SSP, Shared Socioeconomic Pathway.

This study also goes beyond the uncertainty factors related to NDCs assessed in the previous studies^19^, ^20^ to comprehensively assess the following five uncertainty factors (Figure 1): (i) socioeconomic baseline variation; (ii) profile of the pathway to net‐zero targets, such as accelerated, linear, or delayed; (iii) uncertainty in the emissions coverage of net‐zero targets, as it highly determines the time delay between achieving net‐zero CO_2_ emissions and net‐zero GHG emissions; (iv) uncertainty in the mitigation potential of non‐CO_2_ GHG sources; and (v) uncertainty in land‐use sinks (nature‐based CDR). The impact of the uncertainty of the negative emissions from CDR technologies is not analyzed here, given the substantial uncertainty regarding their scalability and how countries intend to use these technologies to meet their net‐zero target.^5^, ^7^

The analysis includes emission projections based on GHG emissions pathways toward countries’ net‐zero targets for the world and regions (with a focus on six major emitting countries) using the integrated assessment model IMAGE.^11^, ^20^ The different uncertainty factors result in many independent scenarios per country and for the world, which are explored in a full‐factor uncertainty analysis on annual projected emissions and abatement costs up to 2050. Subsequently, a single‐factor uncertainty analysis based on different regression approaches is used to quantify the contribution of each factor to the total uncertainty. Finally, a technical feasibility assessment of the scenarios is performed, using dynamic time‐ and pathway‐dependent marginal abatement cost (MAC) curves from the IMAGE model.^21^, ^22^

## METHODS

**FIGURE 2:**
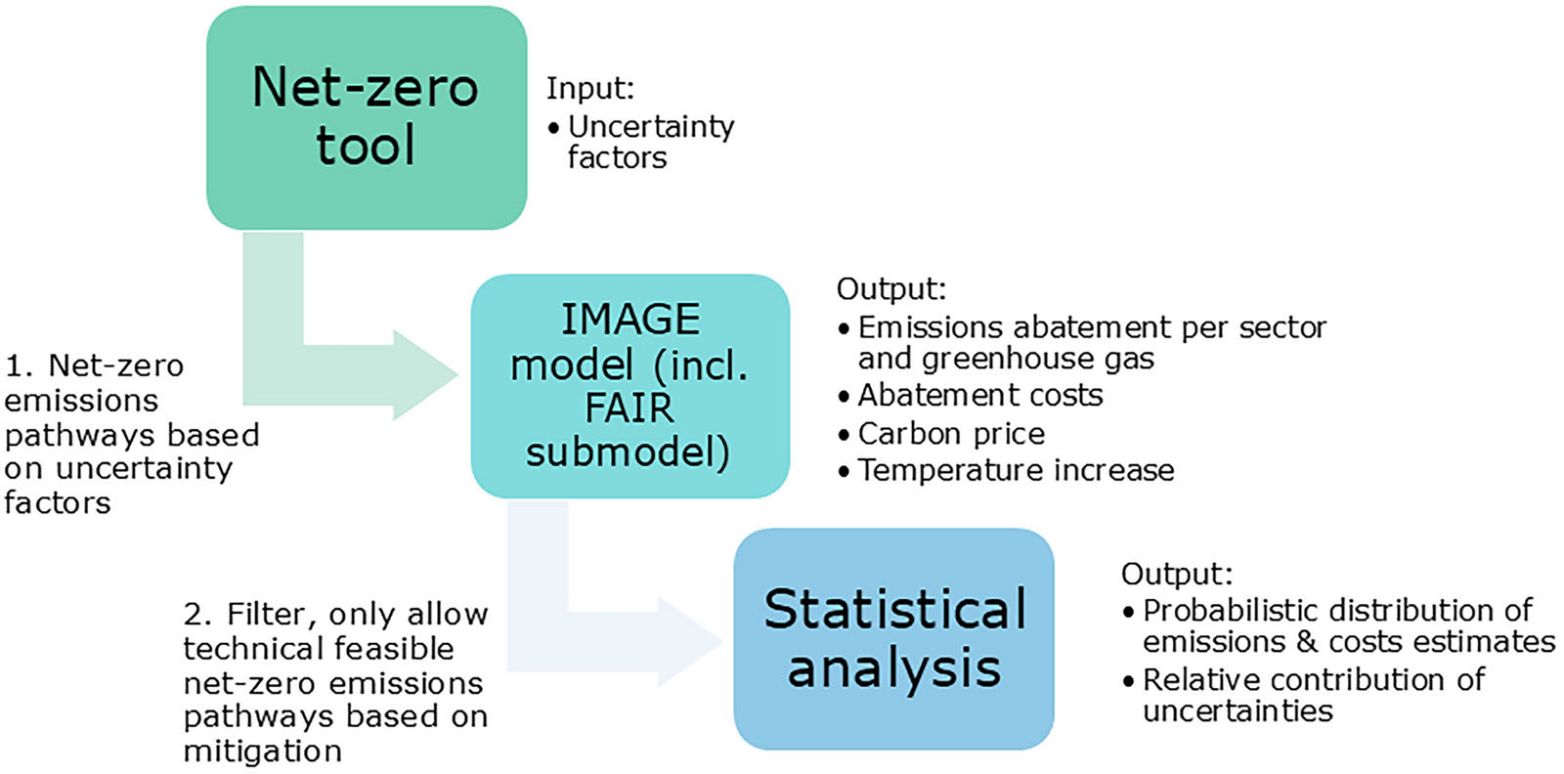
Calculation steps of the analysis of net‐zero targets scenarios.

This study presents the methodology for calculating the corridors of technically feasible GHG emissions pathways toward countries’ net‐zero targets in CO_2_ equivalent terms.^a^ The key results are presented as GHG emissions, excluding and including the land‐use, land‐use change, and forestry (LULUCF) sector, abatement costs, and carbon price projections. The calculations are performed in three steps (see Figure 2).

First, we quantify emissions projections for all possible net‐zero target scenarios, following the combination of uncertainties affecting the net‐zero targets. This analysis includes the full set of GHG emissions pathways implied by 648 net‐zero target scenarios, based on all combinations of uncertainty factor subcategories for the 26 world regions of IMAGE.

Second, we use the IMAGE model and its submodels to calculate the technically feasible GHG emissions pathways and the abatement costs and carbon price projections based on the information on the mitigation potential of all GHG emissions and sources, as well as the temperature increase.

Third, we calculate the probabilistic distribution of emissions and abatement costs based on the calculated feasible pathways from step 2. In addition, we calculate the relative contributions of the uncertainty factors to the total uncertainty ranges of the projections of GHG emissions and abatement costs of the technically feasible pathways.

### Uncertainty factors considered and the estimation of possible country‐level emission trajectories

The selection of net‐zero targets and the information about their formulation for 32 major emitting countries with net‐zero targets (about 80% of global Kyoto GHG emissions in 2019) was determined by consulting three independent net‐zero target trackers^1^, ^23^, ^24^ (Table 1)—a methodology also employed to assess these countries in Rogelj et al.^2^ Regarding the target formulation, we focus on the information around the coverage of gases and the net‐zero target year.

**TABLE 1:** Details of net‐zero target year and GHG coverage for the IMAGE regions.

IMAGE region^a^	Official net‐zero target year	Emission coverage	Main emitting countries with net‐zero target within the region
Canada	2050	GHG	Canada
USA	2050	GHG	USA
Mexico	N.A.	No zero target	Mexico (no net‐zero target)
Rest of Central America	N.A.	N.A.	Costa Rica (2050, GHG)
Brazil	2050	Unclear	Brazil
Rest of South America	N.A.	GHG (∼55% of regional 2019 emissions with net‐zero target 2050)	Argentina (2050, GHG), Chile (2050, GHG), Colombia (2050, GHG)
Northern Africa	N.A.	N.A.	
Western Africa	N.A.	N.A.	Nigeria (2050, GHG)
Eastern Africa	N.A.	N.A. (∼35% of regional emissions with net‐zero target 2050)	Ethiopia (2050, GHG)
South Africa	2050	CO_2_	South Africa
Rest of South Africa	N.A.	N.A.	
Western Europe^b^	2050	GHG	EU27 (2050, GHG), UK (2050, GHG)
Central Europe^b^	2050	GHG	EU27 (2050, GHG)
Turkey	2053	CO_2_	Turkey
Ukraine	2060	GHG	Ukraine
Central Asia	N.A.	GHG (∼50% of regional emissions with net‐zero target 2060)	Kazakhstan (2060, GHG)
Russian Federation	2060	GHG	Russian Federation
Middle East	N.A.	Unclear (∼35% of regional emissions with net‐zero target 2060)	Saudi Arabia (2060, unclear), Israel (2050, unclear), United Arab Emirates (2050, GHG)
India	2070	Unclear	India
Korea region	2050	Unclear	Republic of Korea (2050, unclear)
China region	2060	Unclear	China
Southeastern Asia	N.A.	Unclear (∼70% of regional emissions with net‐zero target 2050)	Cambodia (2050, CO_2_), Malaysia (2050, unclear), Nepal (2045, CO_2_), Thailand (2050, CO_2_), Singapore (2050, GHG), Vietnam (2050, GHG)
Indonesia	2060	Unclear	Indonesia
Japan	2050	GHG	Japan
Oceania	2050	GHG	Australia (2050, GHG) and New Zealand (2050, CO_2_)

*Note*: Source comes from adjusted Dafnomilis et al.^11^, ^13^ and literature sources.^1^, ^23^, ^24^

Abbreviation: GHG, greenhouse gas, N.A., not available

^a^ https://models.pbl.nl/image/Region_classification_map.

^b^ All Member States of the EU27 and the United Kingdom are included in the IMAGE calculations as part of the Western and Eastern European regions; in the results, we present the results for EU27.

### Overview of uncertainty factors

Based on previous research,^2^, ^20^ we identified a set of factors driving uncertainty in the net‐zero targets^b^ (Figure 2), as explained below.

### Gas coverage of net‐zero targets

This is modeled as the assumed time lag in years between achieving net‐zero CO_2_ emissions and net‐zero GHG emissions. For all countries with CO_2_‐only coverage, we assume that net‐zero GHG emissions will be achieved 15 years later, with a range of 10−20 years, except for India and Indonesia, where we consider a time lag of 30 years later with a range of 10−50 years. These time lags are based on the pathways of the major emitting countries, derived from an assessment of cost‐effective 1.5°C and 2°C scenarios from integrated assessment models by van Soest et al.,^25^ and the analysis of C1a and C3 category scenarios presented in the Intergovernmental Panel on Climate Change (IPCC) Sixth Assessment Report.^26^ For countries with unclear coverage of the net‐zero targets, such as Brazil, China, and India, we assume two options: (i) the conservative estimate for CO_2_ only and (ii) the optimistic estimate for all GHGs (see Figure 1). The countries without net‐zero targets follow the current policies trajectory.

### Profile of the emissions pathway

The path taken from current levels to net‐zero targets determines the total amount of CO_2_ and other GHGs emitted and thus the total carbon budget used. The climate outcome is affected by whether a linear, accelerated, or delayed path is followed.^27^ For this study, the four pathways toward achieving the long‐term net‐zero targets include: (i) an accelerated reduction trajectory, which is differentiated reductions between countries based on income by 2030; (ii) a linear path starting in 2023, and a delayed trajectory starting from the 2030 emission levels; (iii) implied by countries’ conditional NDC target; or (iv) current policies in 2030. After 2030, all four pathways follow a linear path toward countries’ net‐zero targets. For the accelerated pathway, we assume that emissions will be reduced by 50% in 2030 relative to 2022 levels for high‐income countries, 30% for middle‐income countries, and 20% for low‐income countries by 2030.

### Socioeconomic baseline variation

This factor mainly affects the delayed net‐zero emissions trajectories starting from current policies by 2030. However, it also affects those departing from 2030 NDC targets that are indexed to socioeconomic developments (such as for China and India).^28^ Socioeconomic baseline uncertainties also influence the emissions projections for countries with no net‐zero targets that follow the current policies’ trajectory, which are based on three socioeconomic futures in 2030.

### Non‐CO_2_ GHG mitigation potential

Non‐CO_2_ GHG emissions reductions affect the feasibility of net‐zero emission pathways. Achieving net‐zero targets will require unprecedented reductions in emissions of CO_2_ as well as non‐CO_2_ gases such as methane, nitrous oxide, and fluorinated gases. CO_2_ has received most of the attention in climate policy research because of its large share of total emissions, but non‐CO_2_ emissions are becoming increasingly important because non‐CO_2_ emissions from the agricultural sector, such as methane from livestock or nitrous oxide from fertilizers, are especially hard to abate.^8^, ^10^, ^29^ However, the exact level of the remaining non‐CO_2_ emissions is highly uncertain. Harmsen et al.^29^ provide a systematic bottom‐up estimate of the total uncertainty in non‐CO_2_ abatement by developing *optimistic*, *default*, and *pessimistic* long‐term MAC curves (i.e., with high, medium, and low reduction potentials, respectively), based on a comprehensive literature review of mitigation options. These MAC curves are used in this study. A higher reduction potential leads to a higher non‐CO_2_/CO_2_ reduction ratio, but it does not affect the net reduction (in CO_2_ equivalents), provided that the net‐zero target is met.

### Land‐use contributions

Historical (and projected) land‐use emissions reported by countries to the UNFCCC generally differ from those used by integrated assessment models. There are conceptual differences in how integrated assessment models and national GHG inventory data define anthropogenic land‐use CO_2_ fluxes due to simplified and/or incomplete representation of forest management in integrated assessment models. There are also differences in how land‐use categories are accounted for, particularly concerning unmanaged land and unmanaged forests. Forsell et al.^30^ have conducted a more detailed analysis of governments’ long‐term strategies submitted to the UNFCCC to examine projections of emissions and sinks from LULUCF in line with the national NDCs^31^ and Long‐term Low Emission Development Strategies commitments^c^ ^,32^ (for further details, see Supporting Information S1). We consider three scenarios for the LULUCF emissions projections: (i) net LULUCF emissions declining over time according to countries’ own estimates as documented in the LULUCF dataset^30^; (ii) LULUCF‐related emissions and sinks projections associated with mitigation options in the LULUCF sector based on the IMAGE model calculations for a 1.5°C scenario^8^, ^13^; and (iii) similar LULUCF emissions and sinks projections, but now based on the IIASA's global land‐use model GLOBIOM^33^ calculations for a 1.5°C scenario.^34^, ^35^ For all three scenarios, the land‐use emissions and sinks projection for the regions are harmonized with national inventory data using the offset method and the offset factor kept constant over time.^30^ The inventory data used to harmonize the projections were collected by Forsell et al.,^30^ using an approach similar to Forsell et al.^36^ and Grassi et al.^37^ (see Supporting Information S1).

### Development of global and regional net‐zero emission trajectories using the IMAGE model

IMAGE is an integrated assessment model framework that simulates global and regional environmental consequences of changes in human activities^22^, ^38^, ^39^ (see also Supporting Information S2). IMAGE is a simulation model (i.e., changes in model variables are calculated based on the information from the previous time step). The model includes a detailed description of the energy and land‐use system and simulates most of the socioeconomic indicators (such as population and income) for 26 regions. The IMAGE model includes a slightly adapted version of the climate model MAGICC 6.3.^40^

Table 1 presents the net‐zero target years and the gas coverage included in each country's net‐zero pledge across the 26 regions. As some IMAGE regions consist of several countries, the calculation of NDC/net‐zero targets becomes more complex when there is no dominant emitter. In regions such as the Middle East, where only some countries have committed to net‐zero targets, the final emissions target for the net‐zero year is estimated based on the share of GHG emissions from countries with net‐zero pledges, while emissions from the remaining countries follow the current policies scenario for the region (see Table 1). The methodology is based on Dafnomilis et al.^11^, ^13^

We use the IMAGE model^22^, ^38^, ^39^ to identify which of the emissions pathways are technically feasible, and to quantify the abatement costs and the carbon price, as well as the allocation of the abatement across the different gases. For this purpose, we included the calculated net‐zero scenarios (in the form of emissions targets, excluding the emissions and removals from LULUCF) in IMAGE, which act as constraints and lead to increased economy‐wide, regional, and country‐specific carbon prices and abatement costs depending on the pathway and the final net‐zero target level.^28^ In the final analysis, only the technically feasible pathways are included. To filter the technically feasible projections, the carbon price is used as a condition. If the carbon price exceeds a certain threshold (1450 (2020)$/ton CO_2_, i.e., the maximum price level in IMAGE) for more than 5 years over the whole period from 2020 to the net‐zero target year, this scenario is removed from the set of scenarios. For this analysis, we consider a scenario infeasible if this happens for at least four of the 26 world regions for at least 5 successive years. The temperature increase projections are calculated using the MAGICC 6.3 climate model based on global GHG emissions, with the global land‐use emissions harmonized with the historical emissions of the IMAGE model.

IMAGE's FAIR model calculates the impact of climate mitigation policy using carbon prices and MAC curves, which represent the costs of mitigation actions to determine a cost‐optimal emission pathway^41^, ^42^ (see also Supporting Information S2). It captures the time‐ and pathway‐dependent dynamics of the underlying energy model of IMAGE by scaling the MAC curves based on the reduction effort of previous years. The MAC curves in FAIR are based on (i) the IMAGE energy model for energy‐related CO_2_ emissions^21^ and (ii) MACs for non‐CO_2_ emissions as described in Harmsen et al.^29^, ^43^; see Hof et al.^38^ The MAC curves for energy‐related CO_2_ emissions were constructed to account for past efforts by imposing a wide range of carbon price pathways in the energy model of IMAGE and capturing the induced reduction in CO_2_ emissions^44^ (see also Supporting Information S2).

### Current policies scenario of IMAGE

The countries without net‐zero targets follow the current policies scenario of IMAGE. More specifically, the effect of climate mitigation policies that have been adopted and implemented as of November 2023 on GHG emissions in all sectors up to 2030 was projected using the integrated assessment model IMAGE,^21^ which includes the energy system model. Current climate and energy policies from G20 economies, as identified in the public database on climate policies,^45^ d the ENGAGE project,^e^ and policy overview updates^46^, ^47^ were added on top of the updated IMAGE SSP2 reference scenario. More specifically, we used a modeling protocol,^48^ updated from Roelfsema et al.,^48^, ^49^ including a detailed spreadsheet listing policies by country to implement current policies in the IMAGE model. For this study, the current policies scenario projections for the IMAGE SSP1 and SSP3 scenarios were calculated using the same methodology.^48^ More specifically, we develop our current policies scenario using three shared socioeconomic pathways (SSPs): SSP1, SSP2, and SSP3. These are developed using the methodology of Roelfsema et al.,^48^ and project GHG emissions and energy use as affected by economic, demographic, and social drivers.^50^ SSP1 represents a sustainable future path in which environmental boundaries are respected.^50^ SSP2 is a middle‐of‐the‐road scenario, which is used as default. In SSP3, environmental issues have low priority and the world is characterized by regional rivalry.^50^ Similar as in the earlier uncertainty studies,^19^, ^20^ we assume that all three socioeconomic scenarios have the same probability despite acknowledging that the SSPs were not originally designed to have assigned probabilities. Extrapolation for the emissions projections beyond 2030 follows the method of van Soest et al.^51^ of extrapolating the equivalent carbon price in 2030 using the GDP growth rate of the different regions and is mostly used for illustration.

### Statistical analysis on the distribution of emission projections

For this analysis, we used the ANOVA variance decompositions to analyze how much of the variability in the output variables depends on the independent variables. This approach also takes into account the interactions between the uncertainty factors. For this study, the ANOVA analysis was used to estimate the relative importance of each uncertainty factor in determining the output variable (i.e., projected GHG emissions and total abatement costs in 2050).

## RESULTS

### Emissions

**FIGURE 3:**
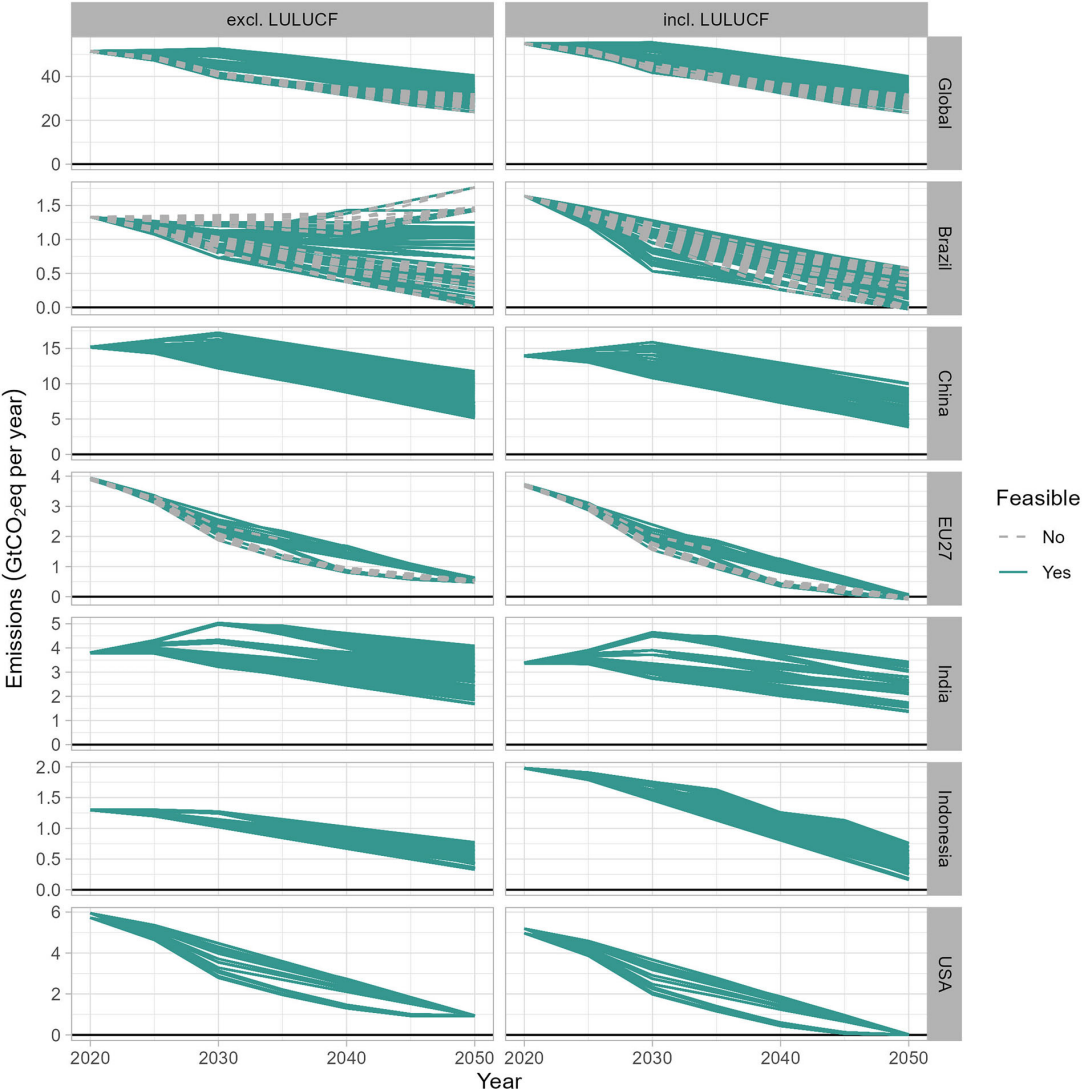
Net‐zero emissions corridors for the world and six major emitters (showing the full and technically feasible ranges). Abbreviation: GtCO_2_eq, gigatons of CO_2_ equivalent.

Figure 3 shows the projected emissions ranges based on all feasible and infeasible pathways until 2050 for the world and the six major emitting countries based on various input factors. The projected emissions ranges of the other world regions are given in Figure S1. The results are based on 648 net‐zero target scenarios per country and the world, representing all possible combinations of uncertainty dimensions.

About 574 scenarios (89% of all generated scenarios) are technically feasible pathways (Figure 3). The other scenarios are found to be infeasible in the IMAGE model. Infeasibilities occur in the model due to a lack of timely mitigation options to meet the reduction targets and can be caused by several reasons, such as relatively high residual non‐CO_2_ emissions, a lack of CDR mitigation options, or binding constraints on the diffusion of mitigation technologies. This is exhibited in the model by the carbon price exceeding a predefined maximum price threshold.

We found that infeasible pathways occur in only about 46% of the accelerated pathways (74 runs out of a total of 162 runs). The infeasibilities occur mainly for EU27 and Canada (on average 15 years above the price threshold) and to a lesser extent for Turkey and Oceania (on average only 5 years), while the accelerated pathways are all feasible for China, India, and the United States.

**FIGURE 4:**
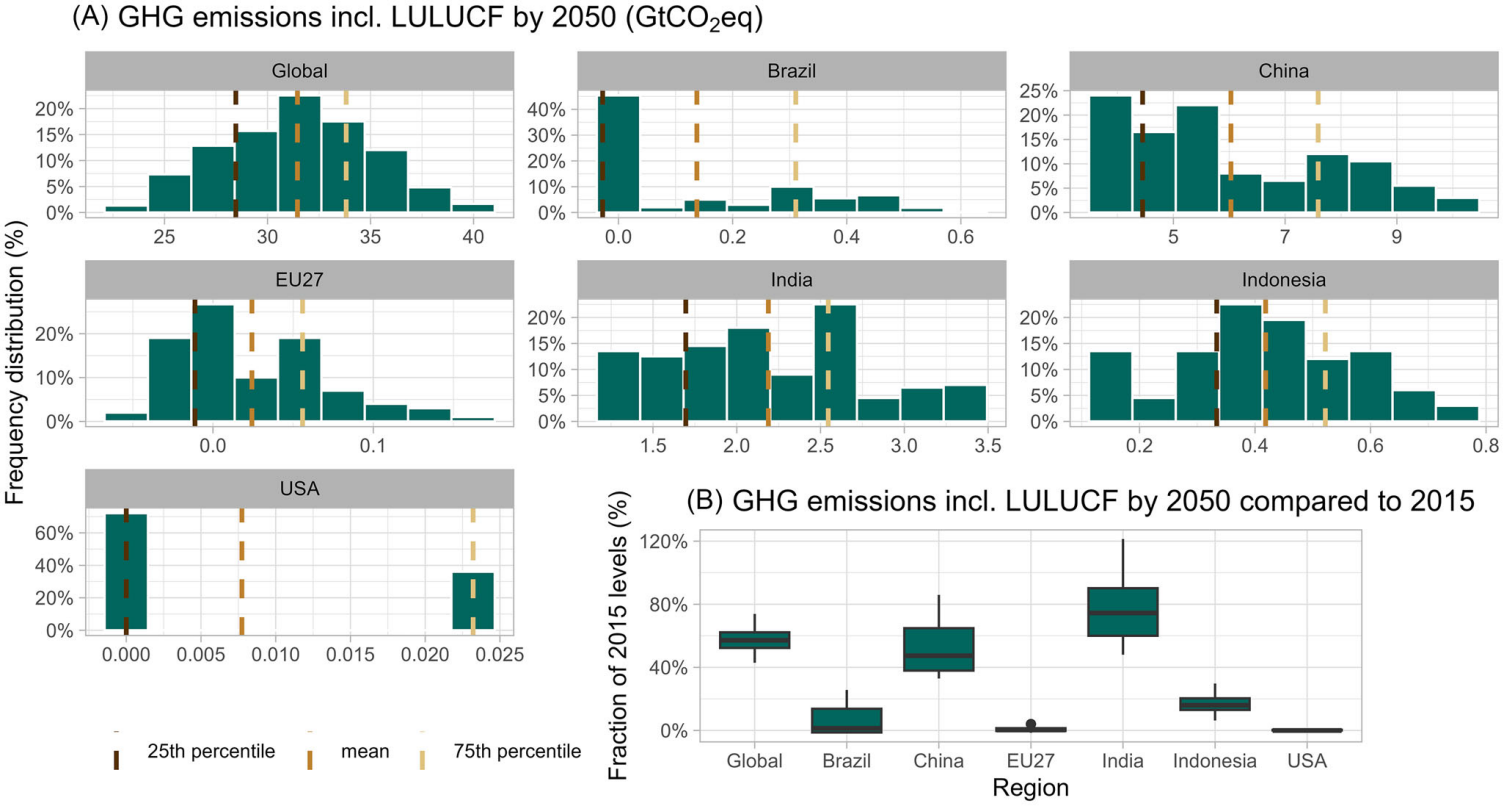
Frequency distribution of 2050 GHG emission projections (including LULUCF) (panel A) for the six major emitters and the world according to net‐zero targets. The figures for the other regions are given in Figure S1. Panel B presents the boxplot diagrams of all GHG emissions relative to 2015 emissions. Abbreviations: GHG, greenhouse gas; GtCO_2_eq, gigatons of CO_2_ equivalent; LULUCF, land‐use, land‐use change and forestry.

LULUCF sinks are important in achieving the net‐zero targets in the target year (compare the left and right columns of Figure 3). GHG emissions show a wide range by 2030 mainly depending on the assumed profile of pathways of countries, varying from a linear, accelerated, or delayed path (NDC or current policies level) toward reaching the net‐zero target. For the EU27 and the United States—which both have net‐zero GHG targets for 2050—the projected GHG emissions pathways including LULUCF converge toward a net‐zero point in 2050. However, the emissions pathway excluding LULUCF result in a range of emissions by 2050 (see also Figure 4). For the United States, the GHG emissions excluding LULUCF in 2050 are mainly residual emissions that need to be compensated by land‐use sinks. The land‐use sinks show a small range from 0.9 to 0.95 GtCO_2_. For the EU27, about 80% of all scenarios are assumed to be technically feasible (i.e., to exceed the price threshold in less than 5 years), which is highly dependent on the low projected uptake of CDR technologies and the limited remaining non‐CO_2_ mitigation potential in the EU27 in our modeling framework. The technically feasible emissions pathways (including LULUCF) reach net‐zero (or close to net‐zero) by 2050 (Figure 4).

In contrast, China, India, Brazil, and Indonesia have net‐zero targets beyond 2050 for which the gas coverage is unclear. Consequently, the emissions pathways of these regions are highly dependent on the assumed time lag in achieving net‐zero CO_2_ and net‐zero GHG emissions, as well as the profile of the pathway. Therefore, we project a higher uncertainty range for these regions for 2050 GHG emissions including and excluding LULUCF. The assumed LULUCF sinks further widen these ranges. More specifically, China's 2050 GHG emissions (incl. LULUCF) reach about 47% of 2015 emissions (median) (Figure 4), with a range of 35−85% (25th to 75th percentile range: 40−65%), while for India's 2050 emissions reach about 75% of 2015 emissions levels (median, with a range of 50−120%). The median 2050 emissions for China depend on LULUCF emissions (i.e., 5.6 GtCO_2_eq) (incl. LULUCF) and 7.1 GtCO_2_eq (excl. LULUCF), which is a difference of 1.5 GtCO_2_eq (for comparison, the difference for India is only 0.5 GtCO_2_eq). For Brazil, the impact of LULUCF emissions is even larger. The median emissions for Brazil in 2050 are almost zero (incl. LULUCF) and 0.9 GtCO_2_eq (excl. LULUCF) (Figure S1), which are 100% and 50% below 2015 levels, respectively. The projections also show a wide range, especially for emissions without LULUCF. For Indonesia, the impact of LULUCF emissions on the 2050 emission projections is much smaller (Figure S1).

An important group are the countries without net‐zero targets, for which current policy emission levels were assumed. The GHG emissions of this group of countries increase from about 8.3 GtCO_2_eq in 2015 to 9.1–14.3 GtCO_2_eq (full range, with a median estimate; 13.1 GtCO_2_eq) in 2050. This range is heavily influenced by the uncertainty in the land‐use emissions and in the current policy projections from the socioeconomic baseline variation. Its share of global emissions increases from about 16% in 2015 to 42% (full range 36–39%) in 2050 for the net‐zero target scenarios (Table S1).

Figure 4 shows the density plots for the projected emissions in 2050 for the major emitting countries and the world. Uncertainties result in global GHG emission projections (including LULUCF) in 2050 ranging from 23 to 40 GtCO_2_eq (40–75% of 2015 emissions levels), with a median estimate of 31 GtCO_2_eq (55% of 2015 levels). The 25th to 75th percentile range is 28−34 GtCO_2_eq.

The resulting global mean temperature increase projections by 2100 range from 1.8°C to 2.2°C, with a median estimate of 2.0°C. Note that this is the climate range under a median climate sensitivity; if we were to present the range under the climate sensitivity range, it would be much wider.

**FIGURE 5:**
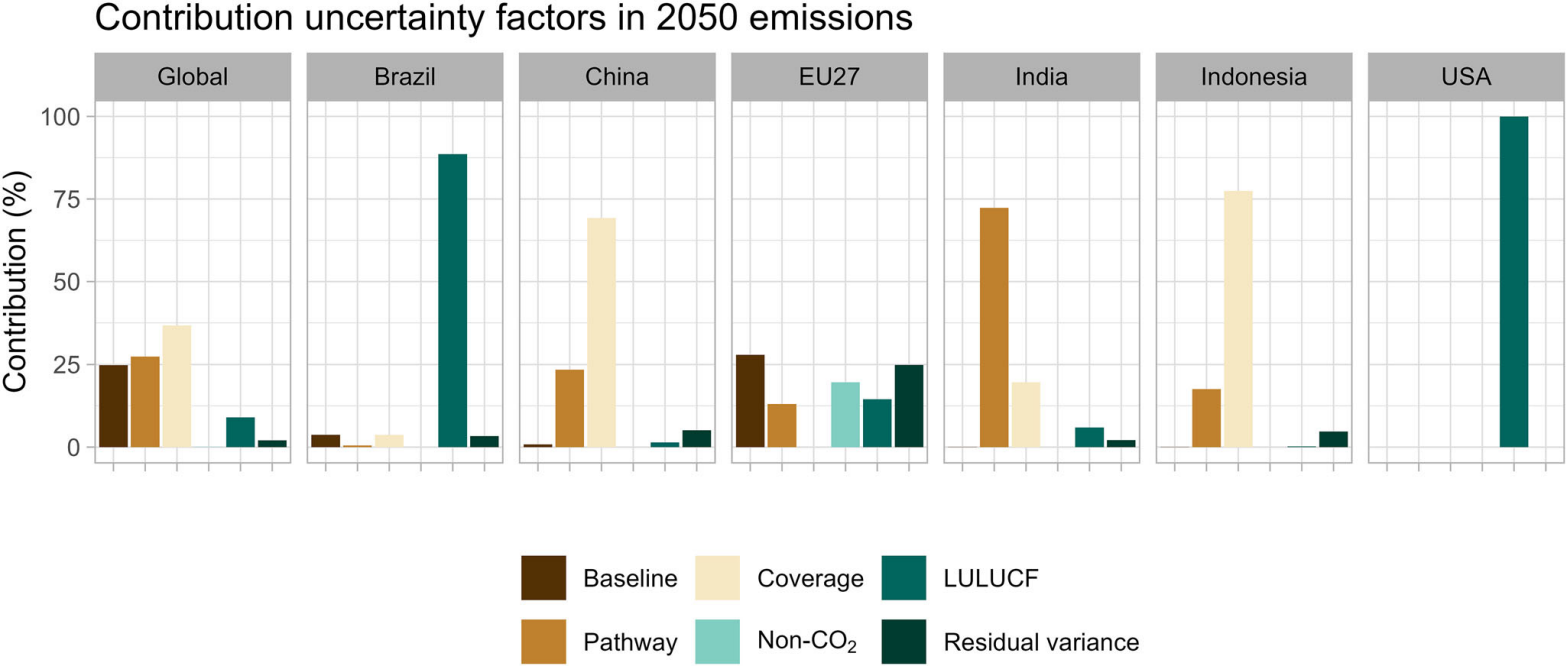
Contribution of uncertainty factors in the 2050 GHG (incl. LULUCF) emission projections. Abbreviation: LULUCF, land‐use, land‐use change and forestry.

There are notable differences between countries considering the factors contributing to the variability of projected emissions in 2050 (Figure 5). For Brazil and the United States, the land‐use category emerges as the most important contributor. For the EU27, the main source of uncertainty is the socioeconomic baseline used, which is largely responsible for the fact that not all pathways are technically feasible to reach the net‐zero target. In contrast, for countries with net‐zero targets beyond 2050, such as China and Indonesia, the net‐zero gas coverage and, to a lesser extent, the pathway profile becomes the dominant category influencing the variability of projected emissions. For India, the pathway is the most important factor.

On a global level, the net‐zero coverage is the most important contributing factor (about 35%). This is followed by two equally important factors: pathway and baseline. In contrast, the uncertainty in non‐CO_2_ mitigation potential has a minimal to negligible influence, as it only affects the CO_2_/non‐CO_2_ ratio and not the net reductions. However, a lower non‐CO_2_ mitigation potential can result in infeasible emission pathways, especially for the EU27, which has a 2050 net‐zero target and limited potential for negative emissions. This is indicated by the small contribution of non‐CO_2_ uncertainty to the EU27 emissions in Figure 5.

### Contribution of the GHGs to total mitigation

**FIGURE 6:**
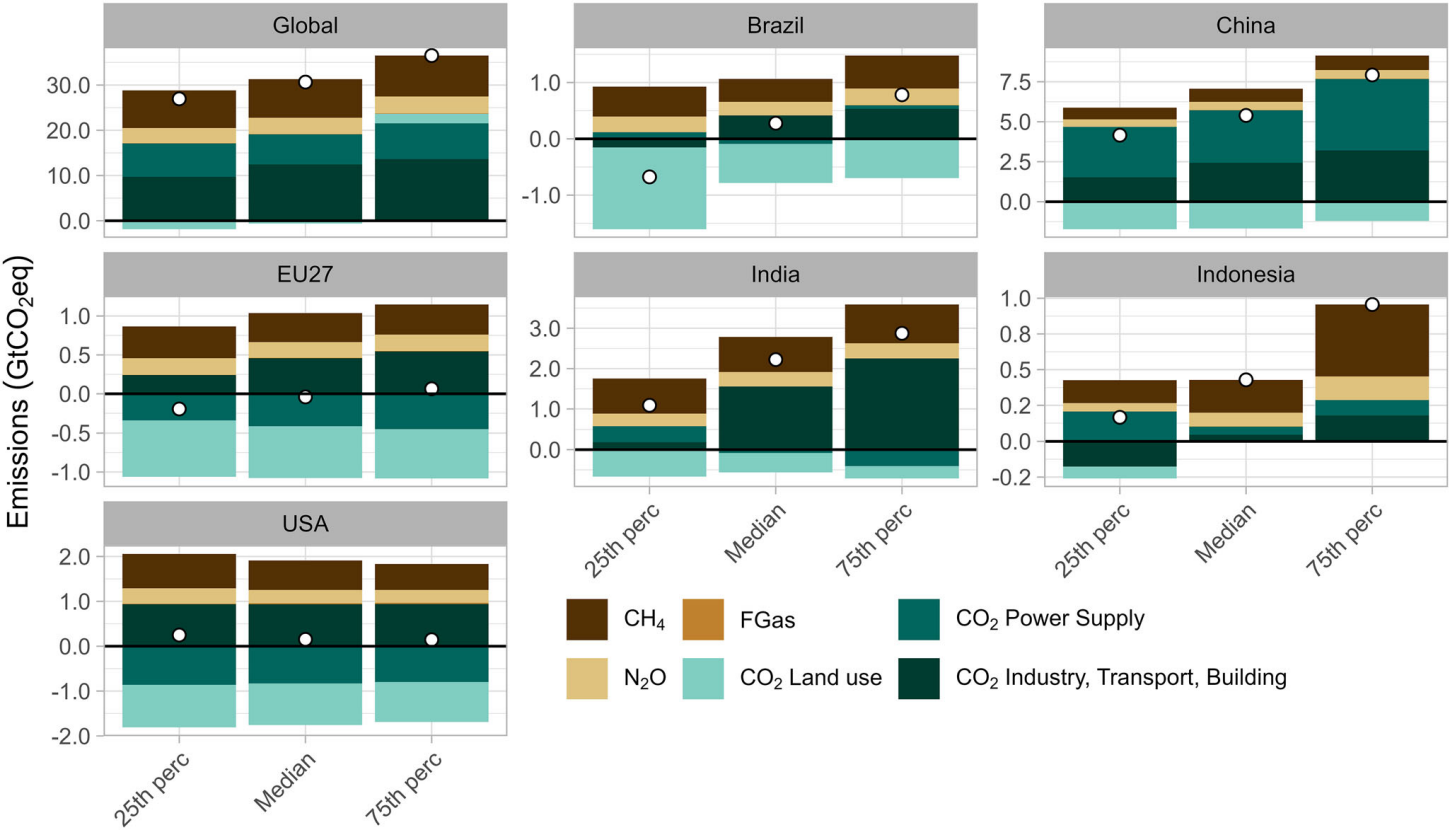
Breakdown of emissions in 2050 of total greenhouse gas emissions based on median, average, and 25th and 75th percentile estimates. The white dots represent the net GHG emissions in 2050. Emissions of GHG, by greenhouse gas (colors) and country (panels). Positive numbers denote remaining emissions of CH_4_, N_2_O, and F‐gases, and of CO_2_ in industry, buildings, and transport and energy supply, whereas negative numbers denote negative emissions in land use. Negative emissions in this sector result from the use of biomass energy with carbon capture and storage. Abbreviations: GHG, greenhouse gas; GtCO_2_eq, gigatons of CO_2_ equivalent.

The distribution of the different GHG emission sources and sinks in 2050 for the selected countries and the world is shown in Figure 6. Global emissions of different GHGs are reduced significantly by 2050 (with land‐use resulting in negative emissions), but residual emissions are still notable. CO_2_ emissions from transport, industry, and buildings are responsible for about 60–65% of residual emissions in 2050, and the remaining comes from non‐CO_2_ emissions. Residual emissions are extensively discussed in the literature.^4^, ^6^, ^8^, ^9^, ^10^, ^52^, ^53^

For the countries with net‐zero targets by 2050 (EU27 and the United States), achieving net‐zero means that the remaining emissions (methane and N_2_O) are compensated by negative emissions. For the EU27 and the United States, land‐use sinks are the largest contributors to negative emissions, with small variations, and the net‐zero emissions are achieved for the 25th to 75th percentile range of projections (Figure 6). For the countries with a net‐zero GHG year after 2050, but without a specified GHG coverage (China, India, and Brazil), the net GHG emissions by 2050 show a wide range. This variation is mainly due to the variations in the projected CO_2_ emissions from transport, industry, and buildings and the land use sinks (notably Brazil), and to a lesser extent from variations in the methane and N_2_O emissions (Figure 6). For China, CO_2_ emissions from transport, industry, and buildings account for most of the residual emissions. For India, the transport, industry, and buildings sectors slightly dominate the non‐CO_2_ emissions in the residual emissions, while for Brazil, the methane emissions from agriculture are the most important source of residual emissions.

### Abatement costs

**FIGURE 7:**
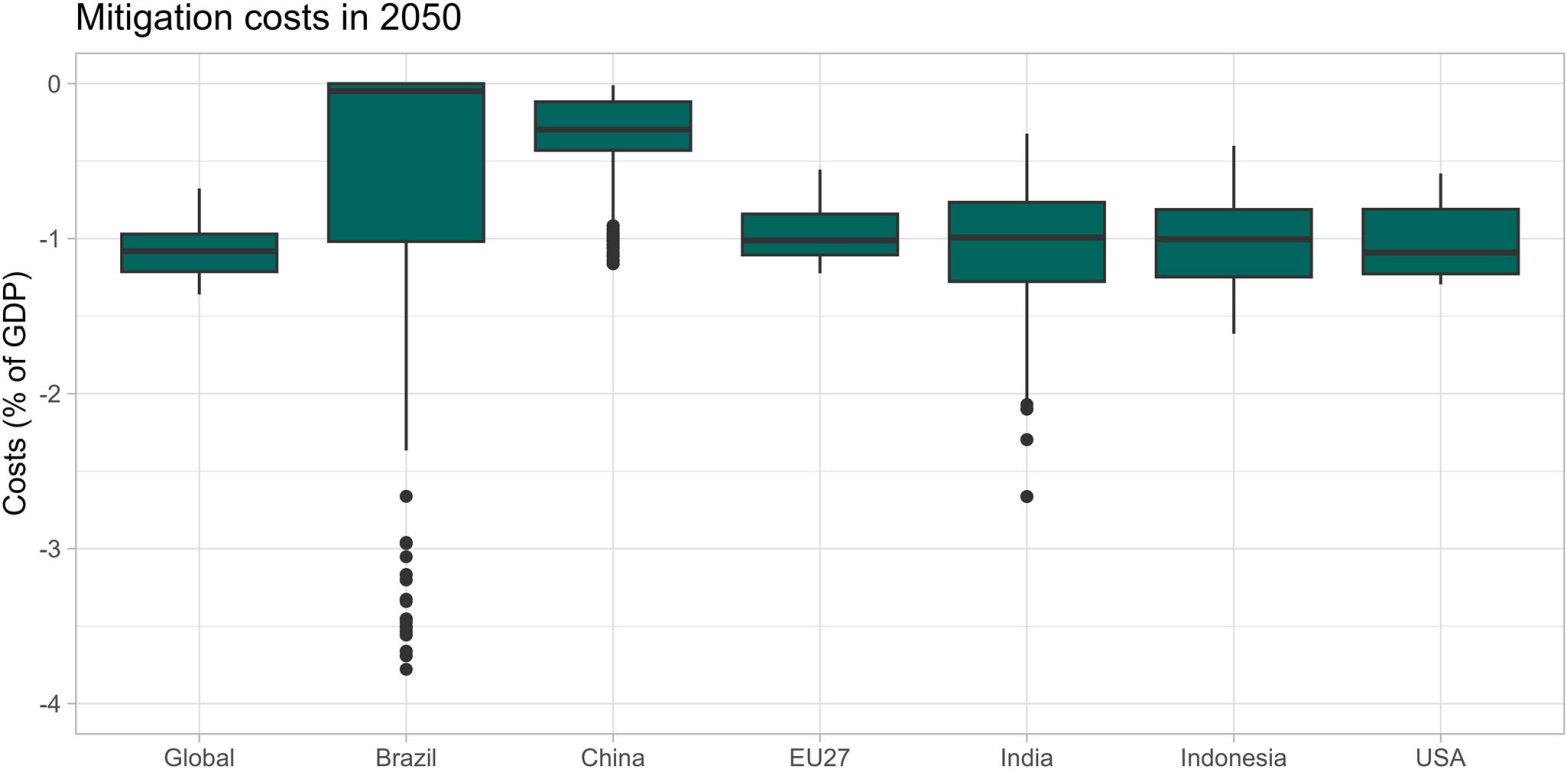
Box plots of 2050 abatement costs projections for Brazil, China, EU27, India, Indonesia, the United States, and the world according to net‐zero targets. Abbreviation: GDP, gross domestic product.

The global abatement costs in 2050 range from −0.7% to −1.4% of GDP (median estimate of −1.1%). Figure 7 illustrates the box plots of projected abatement costs as a percentage of GDP for the major emitting countries (Table S2 gives the detailed data). For the United States, abatement costs amount to −0.8% to −1.2% of GDP (25th to 75th percentile range), with a wide uncertainty range of the costs attributable to SSP scenarios. For the EU27, the abatement costs for the feasible pathways amount to −0.8% to −1.1% of GDP, which are slightly lower compared to the United States, however, for the infeasible pathways are higher. China has lower abatement costs (−0.1% to −0.4% of GDP). India has a range of abatement costs (−0.8% to −1.3% of GDP).

**FIGURE 8:**
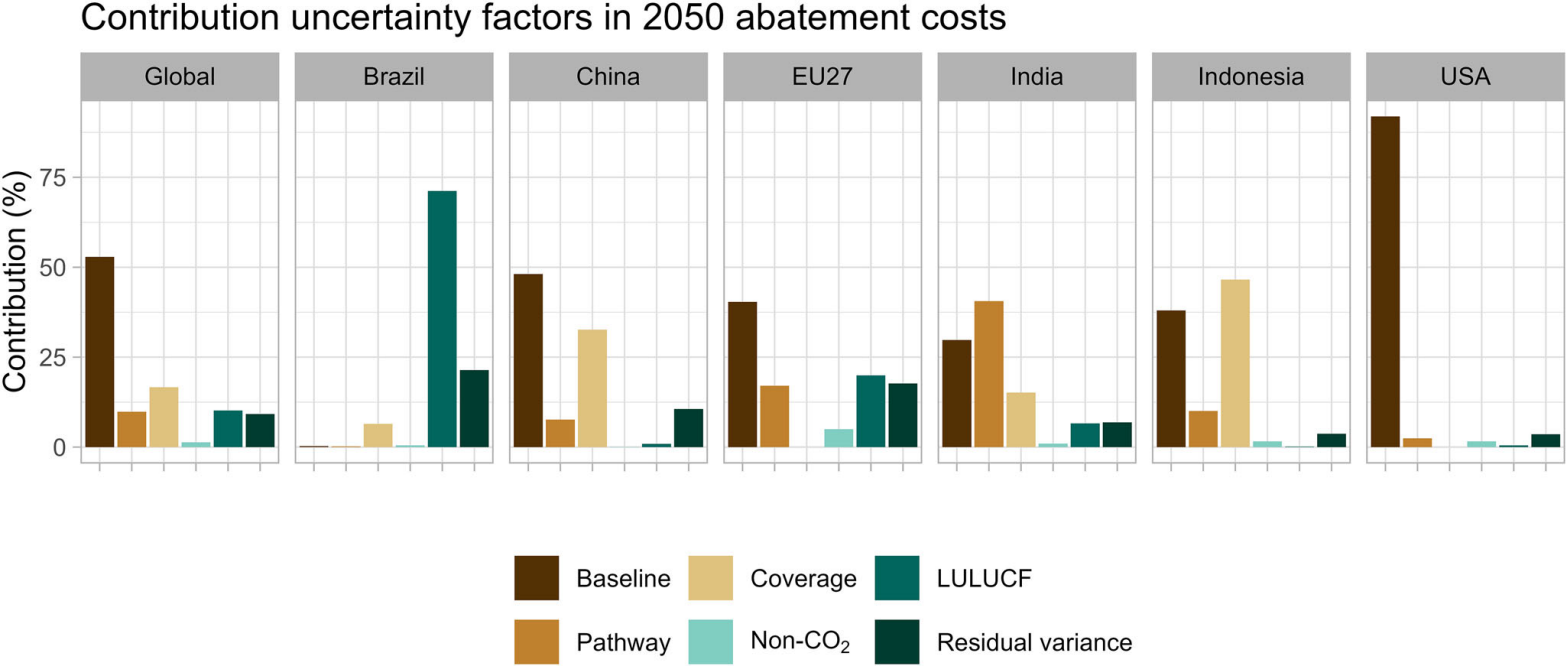
Contribution of uncertainty factors in the 2050 abatement costs.

Looking at the factors contributing to the variability of projected abatement costs in 2050, as calculated by the variance decomposition method, we see that the baseline contributes the most to the uncertainty for all countries, except for Brazil with LULUCF as the dominant factor (Figure 8). The socioeconomic baseline and the profile of the pathway also dominate the uncertainty in global abatement costs.

## DISCUSSION AND CONCLUSIONS

### Significance of the study and key findings

We used an integrated assessment model to analyze the impact of key uncertainties associated with countries’ recent net‐zero targets on GHG emission projections for major emitting countries and the world. To the authors’ knowledge, there is no systematic uncertainty analysis of how key uncertainties associated with net‐zero targets affect projected global emissions.

At the global level, these uncertainties lead to projections of GHG emissions (including LULUCF) in the range 23–40 GtCO_2_eq in 2050 (full range, with a median estimate of 31 GtCO_2_eq). Our full range corresponds to 40–70% of 2015 emission levels, which is much wider than the range of 30–45% reported in the multimodel study.^15^ The main sources of uncertainty are, in order of global importance: uncertainty in the net‐zero gas coverage to achieve net‐zero CO_2_ and GHG emissions, uncertainty in the socioeconomic baseline, and uncertainty in the profile of the emission pathway to achieve net‐zero targets.

At the country level, for the countries with net‐zero 2050 GHG targets (Canada, the EU27, and the United States), their GHG emission projections to 2050 show a small range, and the main source of uncertainty is land use emissions for the United States and Canada and the socioeconomic baseline for the EU27. In contrast, countries with net‐zero targets beyond 2050 and unclear coverage, such as China, India, and Indonesia, have a wide range of 2050 GHG emissions. China's 2050 emissions (including LULUCF) range from 35% to 85% (full range, median: 47%) of 2015 levels by 2050, with the net‐zero gas coverage, to a lesser extent, the pathway, being the main sources of uncertainty. India's 2050 emissions range from 50% to 120% (median: 75%) of 2015 levels, with the pathway and, to a lesser extent, the net‐zero gas coverage now being the main sources of uncertainty. Emissions from countries without net‐zero targets show an increasing trend from around 8.3 GtCO_2_eq in 2015 to 13.1 GtCO_2_eq (full range: 9.1–14.3 GtCO_2_eq) by 2050, and their share of global GHG emissions rises from 16% in 2015 to 42% (full range 36–39%) in 2050. In these cases, the socioeconomic baseline strongly influences the uncertainty range.

### Comparison of the results with previous studies

Several models have also calculated the climate and emissions projections of net‐zero scenarios based on their central assumptions for the countries’ net‐zero targets and have not explored various uncertainties around net‐zero targets based on the updated 2030 pledges and net‐zero pledges as of May, September, and November 2021.^12^, ^16^, ^17^ In addition, Meinshausen et al.^12^ present the global GHG emission pathways for some sensitivity cases for 2030 and long‐term targets, leading to projected global 2050 emissions (excluding LULUCF) of 28–34.5 GtCO_2_eq. In the multimodel comparison study by Tagomori et al.^15^ with six integrated assessment models,^54^ global emissions and climate projections were presented and showed a range of global emissions in 2050 of 17–29 GtCO_2_eq, but part of the uncertainty range is caused by the range of 2015 emissions of 49–57 GtCO_2_eq. Their range is about 30–45% of 2015 emissions levels based on multimodels, which is much lower than our range of 40–70% of 2015 emissions levels. Our larger range is due to the inclusion of three major uncertainty factors (Figure 5): socioeconomic baseline variation, uncertainty in the gas coverage of net‐zero targets (and its related time‐lag between reaching net‐zero CO_2_ and net‐zero GHG emissions), and the profile of the pathway to net‐zero. In contrast, Tagomori et al.^15^ made specific choices regarding the baseline, gas coverage, and pathway profile. In summary, our study agrees with the existing literature that there is considerable uncertainty about the projected emissions implied by net‐zero targets. However, we find an even wider range due to a more explicit consideration of the underlying uncertainties.

### Methodological limitations of the study

There are several research limitations and caveats to the results presented. First, the importance of specific uncertainty factors in the overall uncertainty projections depends strongly on the parameterization of each factor. For example, the impact of the net‐zero gas coverage, modeled as time lag in reaching net‐zero CO_2_ and GHG, depends on the scenarios in the IPCC Sixth Assessment Report's scenarios database. Information from national net‐zero scenarios from countries is limited, especially from countries that have not fully specified the GHG coverage of their net‐zero targets (such as China and India). In addition, the impact of socioeconomic variation depends on the selected SSP scenarios (SSP1–3), but these ranges represent a probabilistic quantification of uncertainty. In addition, the research relies on specific uncertainty factors identified in the study, such as socioeconomic baseline variation and emission reduction pathways. Other factors not included in the analysis could also affect net‐zero projections, such as international offsets covering international shipping and aviation, and separate removal targets, but for many countries, national documents provide no or incomplete information.^1^, ^23^, ^24^

Second, for countries without net‐zero targets, current policy emission levels have been assumed. As a result, their projected emissions to 2050 are heavily influenced by the uncertainty in the socioeconomic baseline changes. Although these countries account for almost 40% of global emissions, their range differs by only 1.8 GtCO_2_eq, which is significantly smaller than the global range of 14.5 GtCO_2_eq. This study does not fully explore the uncertainty ranges of the current policy projections, as these depend heavily on the assumptions made beyond 2030.

Third, we have used one integrated assessment model in this analysis. Some of the uncertainty factors, in particular the short‐term emissions trends on the profile of the pathways (NDC and current policies projections), depend on model assumptions.^2^, ^20^ However, model comparison studies of the NDC and current policy projections have shown that the IMAGE model is well within the range of the other models,^15^, ^49^, ^55^, ^56^ and the range of emissions in 2050 is less dependent on uncertainty in the pathways. However, the technical feasibility of the net‐zero scenarios presented depends on the technology assumptions of the IMAGE model. Almost 90% of all scenarios are considered feasible under the default parameters. Other models or scenario assumptions can be used to identify pathways with higher or lower reliance on CDR and renewable energy technologies and energy demand in the demand sectors, which would affect the feasibility of the net‐zero scenarios.^57^ In the IMAGE results, most of the emission reductions are achieved through a large phase‐out of traditional fossil fuels and a parallel fuel shift to renewables.^22^ However, the persistence of non‐CO_2_ gases and some hard‐to‐abate CO_2_ sectors, as well as limitations on near‐term emission reductions, may require CDR technologies,^8^ and technological limitations in the availability of CDRs in IMAGE lead to infeasible scenarios.

### Policy implications and recommendations

Our results also have implications for the Global Stocktake process as they indicate policy measures to reduce uncertainties in the next round of long‐term targets. Net‐zero targets are very promising because even considering the uncertainty, they result in global emissions by 2050 of 40–70% of 2015 emission levels and the Paris climate goals come into reach (e.g., see Refs. 2, 12, 13, and 17). However, they still result in substantial uncertainty globally. This means that there are substantial risks in discussing climate outcomes with certainty. Making some of these uncertainties explicit helps us identify strategies to minimize them and ensure that net‐zero targets guide actual deep emissions reductions.

Some of these uncertainties are outside the scope of national action, such as the baseline uncertainty. These relate more to how the world as a whole evolves. A world with more competition for resources–like the one we see forming now–would result in emissions at the higher end of the uncertainty range. Realizing emissions pathways in line with the lower end of our uncertainty requires SSP1 “Taking the green road” narrative.

However, several others are within the scope of unilateral decisions, such as addressing scope uncertainty and the reduction pathway. Addressing the uncertainty in the scope of some of these targets, especially those for key emerging economies, is a clear way to reduce uncertainty at the global level. This should be the first step, as it does not require additional action and improves overall transparency to the international community. Accelerating emissions reductions is also fundamental to reducing uncertainty globally. This is an important uncertainty because it is linked with the actual implementation of the net‐zero targets. Deep emissions reductions in the coming decade will not only reduce long‐term uncertainty but also have a substantial impact on climate outcomes and is key to keep the climate goal of the Paris Agreement within reach.

Finally, our research found that many scenarios are considered feasible. This shows that net‐zero targets are already feasible with our current understanding of technology progress.

## AUTHOR CONTRIBUTIONS

All authors contributed to parts of the research and analysis. M.G.J.d.E., I.D., L.N., N.F., M.H., and T.K. conceived and designed the experiments. M.G.J.d.E., I.D., A.B., J.G., and M.H. performed the experiments. M.G.J.d.E., I.D., L.N., A.B., N.F., M.H., and E.H. analyzed the data. M.G.J.d.E., I.D., L.N., A.B., J.G., N.F., M.H., E.H., and Z.A.G. contributed materials/analysis tools. M.G.J.d.E. led the analysis, modeling, and writing of the manuscript. M.G.J.d.E, I.D., L.N., N.F., M.H., and T.K. wrote the paper.

## COMPETING INTERESTS

The authors declare no competing interests.

### Peer review

The peer review history for this article is available at: https://publons.com/publon/10.1111/nyas.15285

## Supporting information



Supporting Information

## Data Availability

The data that support the findings of this study are available from the authors upon reasonable request.

## Footnotes

a All GHG emission figures are expressed using the 100‐year global warming potential (GWPs) from the IPCC Fourth Assessment Report.

b We have not analyzed the impact of the historical emissions uncertainties. Although differences in historical emissions datasets lead to considerable uncertainty in the starting levels of the emissions pathways, their impact on the long‐term (2050) emissions is limited because countries are assumed to be on a net‐zero path and differences in starting levels are assumed to converge to zero by 2100.

c To facilitate the progress toward the global climate goal of the Paris Agreement, countries are invited to document and submit their pledged contributions in the form of NDCs (Article 4.2) and the Long‐term Low Emission Development Strategies (Article 4.9).

d
www.climatepolicydatabase.org

e
https://engage‐climate.org/project/
