# Supplementary material for: Uncertainties around net‐zero climate targets have major impact on greenhouse gas emissions projections
Source: Ann N Y Acad Sci. 2025 Jan 12;1544(1):209–22. doi: 10.1111/nyas.15285 (PMC11829319; doi:10.1111/nyas.15285)
Supplement: Supplementary file 1 — Supporting Information [file NYAS-1544-209-s001.docx]

Supporting Information: Uncertainties around net-zero climate targets have major impact on emissions and climate projections

S1. Description of LULUCF data

LULUCF projections of emissions and removals are harmonized with national GHG inventory data collected from countries GHG inventories in this assessment ^23^. All countries that are part of the United Nations Framework Convention on Climate Change (UNFCCC), are required under Article 4 to develop periodically update, publish, and make available national inventories of anthropogenic emissions by sources and removals by sinks of all GHGs. The dataset includes the national GHG Inventory (NGHGI) data for all UNFCCC countries except the Holy See and thus encompass a total of 195 countries.

For Annex I countries, the NGHGI database was built upon the GHG inventories submitted by countries in their Fifth Biennial Report in 2023. The data was collected from the Common Tabular Format (CTF) tables and is therefore fully consistent in terms of reporting between countries. The reporting timeframe is from 1990 to 2021 and mainly follows the IPCC 2006 Guidelines for NGHGIs.

In contrast, historical GHG data from Non-Annex I is not reported in a standard set of tables as is the case for Annex-I countries, making the data collection slightly more challenging. As established in the Convention, reporting depends on the country's capabilities and the level of international assistance received, which can affect the reporting year. The earliest GHGI from a Non-Annex I country dates back to 2009, while the latest is from 2023.

Regarding the methodologies used for reporting, there is wide range of IPCC methodologies. The recommended methodology is IPCC 1996, however, some countries have adopted more recent methodologies, such as the 2003 Good Practice Guidance for LULUCF or 2006 IPCC Guidelines.

The data collected indicate that the LULUCF sector contributed 3,591 MtCO_2_e of sequestration globally in 2020. This can be compared to global bookkeeping models that estimate net LULUCF CO_2_ emissions at approximately 4,060 MtCO_2_e ^24^. The difference between the two estimates arises from variations in system boundaries, underlying assumptions, and data sources^25-27^.

S2. Supplementary Methods

## Description of scenarios

We use a set of scenarios that investigates a set of simple propositions to align the current national policies and pledges.

The *net-zero targets scenario* assumes full implementation of net-zero targets (cut-off date: January 2024) (Table 1). We assume four pathways to the net-zero targets (see Methods): an accelerated path, a linear path from 2022, or a more delayed path, starting from the conditional NDC target in 2030, or current policies until 2030. Note that for the linear pathway, the NDC targets are not implemented in 2030, unless the NDC target was already more ambitious than the direct to net zero pathway, as was the case for the US and the EU. The calculations focus only on technically feasible scenarios, and to filter these feasible projections, the carbon price is used as a condition. If the carbon price exceeds a certain threshold for more than five years over the entire period from 2020 to the net-zero target year, that scenario is removed from the set of scenarios.

*Current policies scenario of IMAGE.* The effect of climate mitigation policies that have been adopted and implemented as of November 2023, on GHG emissions in all sectors up to 2030 was projected using the integrated assessment model IMAGE ^1^, which includes the TIMER energy system model. Current climate and energy policies from G20 economies, as identified in the public database on climate policies ^2^^[[1]](#footnote-2)^, the ENGAGE project and policy overview updates^3, 4^, were added on top of the updated IMAGE SSP2 reference scenario. More specifically, we used a modeling protocol ^5^, updated from Roelfsema et al. ^5, 6^, including a detailed spreadsheet listing policies by country to implement current policies in the IMAGE model. The *Current policies* scenario also considers the short-term (2020–2025) economic projections updated to include the implications of the COVID-19 pandemic, including changes in sectoral activity ^7^. For this study, the current policies scenario projections for the IMAGE SSP1 and SSP3 scenarios were calculated using the same methodology ^5^. Extrapolation for the emissions projections beyond 2030 follows the method of van Soest et al. ^8^ of extrapolating the equivalent carbon price in 2030, using the GDP growth rate of the different regions and is mostly for illustration.

## Model description

We applied the **IMAGE** integrated assessment modelling framework, version 3.2, to explore the implications of environmental consequences of human activities worldwide ^9-12^. IMAGE includes a detailed description of the energy and land-use system and simulates most of the socio-economic indicators (such as population and income) for 26 major economies and world regions and most environmental indicators based on a geographical grid. The IMAGE modelling framework includes a detailed energy-system model (TIMER), a global climate policy model (FAIR), and a land-use model (IMAGE land).

The **FAIR** model of IMAGE calculates the impact of climate mitigation policy using carbon prices and marginal abatement cost curves (MACs) representing costs of mitigation actions to determine a cost-optimal emission pathway ^13, 14^. It captures the time- and pathway dependent dynamics of the underlying TIMER model by scaling the marginal abatement costs (MAC) curves based on the reduction effort from the previous years. The marginal abatement curves costs curves in FAIR are based on (i) the IMAGE energy model TIMER for energy-related CO_2_ emissions ^1^ and (ii) MACs for non-CO_2_ GHG emissions as described in Harmsen et al. ^15^. Some recent updates were made based on Harmsen et al. ^16^ and EPA ^17^; see Hof et al. ^9^ for more detail. The non-CO_2_ MAC curves are made consistent with the IMAGE scenarios. The MAC curves for energy-related CO_2_ emissions were constructed to account for past efforts by imposing a wide range of carbon price pathways in the TIMER model and recording the induced reduction in CO_2_ emissions ^18^.

The **TIMER** energy model of IMAGE has been developed to explore scenarios for the energy system ^11, 12, 18^. TIMER describes 12 primary energy carriers in 26 world regions and analyses long-term energy demand and supply trends. It covers a wide range of mitigation options, including nuclear power, renewable energy (different solar and wind technologies, hydropower), bioenergy (first and second-generation), nuclear power and CCS technology ^11^. The TIMER model dynamics are mainly determined by the substitution processes of various technologies based on long-term prices and fuel preferences. These two factors drive multinomial logit models that describe investments in new energy production and consumption capacity. The demand for new capacity is limited by assuming that capital goods are replaced not sooner than at the end of their economic lifetime (which is influenced by the carbon price). The long-term prices that drive the model are determined by resource depletion and technology development, which determine the long-term prices that drive the model. Resource depletion is represented by long-term cost-supply curves and technology development by endogenous learning curves or exogenous assumptions. Emissions from the energy system are calculated by multiplying energy consumption and production flows by emission factors. A carbon price can be used to induce a dynamic response, such as the increased use of low- or zero-carbon technologies, energy efficiency improvements, and end-of-pipe emission reduction technologies.

The **land-use model** of IMAGE has been developed to explore scenarios for the land-use system. In terms of land-based mitigation options, IMAGE accounts for three general types of options: bio-energy production, REDD (avoided deforestation) and reforestation of degraded forests. Bio-energy demand is determined by TIMER based on bio-energy yield, the carbon price, dynamics in the energy system, and land availability, following a food-first principle ^19^.

In IMAGE, the main interaction with the earth system is by changes in energy, food and biofuel production that induce land-use changes and emissions of carbon dioxide and other GHGs. The calculated emissions of GHGs and air pollutants are used in IMAGE to derive changes in concentrations of GHGs, ozone precursors and species involved in aerosol formation on a global scale. Climatic change is calculated as global mean temperature change using a slightly adapted version of the MAGICC 6.3 climate model ^20^.

MAGICC’s climate core is based on a 50-layer, hemispherically resolved upwelling-diffusion-entrainment ocean model coupled to a four-box (hemispheric land/ocean) spatial resolution for effective radiative forcing and as such includes enhanced representations of time-varying climate sensitivities, carbon cycle feedbacks, aerosol forcings and ocean heat uptake characteristics. The model runs on monthly timesteps, which improves its representation of the response to volcanic eruptions compared to an annual timestep. The key updates in the most recent version of MAGICC are the inclusion of a state-dependent climate feedback factor (previously it was only forcing-dependent) which has been calibrated to CMIP6 models, accounting for the effect of large historical anthropogenic biomass burning aerosol precursor emissions on aerosol effective radiative forcing, a nitrate aerosol forcing scheme which accounts for the sulphate competition for ammonia based on Hauglustaine et al. ^21^ and the inclusion of a non-ocean heat uptake parameterisation which represents land surface and cryosphere heat uptake in each hemisphere. In addition, it includes an updated effective radiative forcing parameterisations for CO_2_, CH_4_ and N_2_O, while allowing for a wider range of input concentrations ^20, 22^.

S3. Supplementary Tables

Supplementary Table S1: GHG emissions (GtCO_2_eq) (excl. LULUCF) and shares of GHG emissions for the world and five major emitters to net-zero targets for 2050 from the full-factorial analysis.

|  | World | Brazil | China | EU-27 | India | Indo-nesia | USA | Countries without net-zero targets |
| --- | --- | --- | --- | --- | --- | --- | --- | --- |
| Number of feasible scenarios | 574 | 477 | 648 | 534 | 648 | 648 | 648 | 574 |
| *Emissions in GtCO_2_eq* | | | | | | | | |
| Mean | 31.6 | 0.9 | 7.6 | 0.5 | 2.7 | 0.5 | 0.9 | 11.6 |
| Standard deviation | 3.5 | 0.5 | 1.8 | 0.0 | 0.6 | 0.1 | 0.0 | 1.0 |
| minimum | 23.7 | 0.0 | 5.1 | 0.4 | 1.7 | 0.3 | 0.9 | 10.4 |
| 25^th^ percentile | 29.0 | 0.7 | 6.0 | 0.5 | 2.2 | 0.4 | 0.9 | 10.4 |
| Median | 31.4 | 1.0 | 7.1 | 0.5 | 2.7 | 0.4 | 0.9 | 11.7 |
| 75^th^ percentile | 34.1 | 1.4 | 9.2 | 0.5 | 3.1 | 0.6 | 0.9 | 12.7 |
| Maximum | 40.5 | 1.8 | 11.8 | 0.6 | 4.1 | 0.8 | 1.0 | 12.9 |
| *Shares in Global GHG emissions* | | | | | | | | |
| Mean | 100% | 3% | 24% | 2% | 8% | 2% | 3% | 37% |
| Standard deviation | 100% | 14% | 52% | 1% | 17% | 4% | 0% | 30% |
| minimum | 100% | 0% | 22% | 2% | 7% | 1% | 4% | 44% |
| 25^th^ percentile | 100% | 3% | 21% | 2% | 8% | 1% | 3% | 36% |
| Median | 100% | 3% | 23% | 2% | 8% | 1% | 3% | 37% |
| 75^th^ percentile | 100% | 4% | 27% | 2% | 9% | 2% | 3% | 37% |
| Maximum | 100% | 4% | 29% | 1% | 10% | 2% | 2% | 32% |

Supplementary Table S2: GHG emissions (GtCO_2_eq) (incl. LULUCF) and shares of GHG emissions for the world and five major emitters to net-zero targets for 2050 from the full-factorial analysis.

|  | World | Brazil | China | EU-27 | India | Indo-nesia | USA | Countries without net-zero targets |
| --- | --- | --- | --- | --- | --- | --- | --- | --- |
| Number of feasible scenarios | 574 | 477 | 648 | 534 | 648 | 648 | 648 | 574 |
| *Emissions in GtCO_2_eq* | | | | | | | | |
| Mean | 31.5 | 0.1 | 6.0 | 0.0 | 2.2 | 0.5 | 0.6 | 13.0 |
| Standard deviation | 3.4 | 3.4 | 3.4 | 0.1 | 0.6 | 0.1 | 0.0 | 1.0 |
| Minimum | 23.3 | 0.0 | 3.9 | -0.1 | 1.4 | 0.3 | 0.0 | 9.1 |
| 25^th^ percentile | 28.5 | 0.0 | 4.5 | 0.0 | 1.7 | 0.3 | 0.0 | 9.2 |
| Median | 31.1 | 0.0 | 5.6 | 0.0 | 2.1 | 0.4 | 0.0 | 13.1 |
| 75^th^ percentile | 33.8 | 0.3 | 7.6 | 0.1 | 2.5 | 0.6 | 0.0 | 14.1 |
| Maximum | 40.2 | 0.6 | 10.1 | 0.2 | 3.4 | 0.8 | 0.0 | 14.3 |
| *Shares in Global GHG emissions* | | | | | | | | |
| Mean | 100% | 0% | 23% | 0% | 8% | 2% | 3% | 41% |
| Standard deviation | 100% | 100% | 100% | 3% | 17% | 4% | 0% | 31% |
| Minimum | 100% | 0% | 20% | 0% | 7% | 2% | 0% | 39% |
| 25^th^ percentile | 100% | 0% | 19% | 0% | 7% | 1% | 0% | 32% |
| Median | 100% | 0% | 22% | 0% | 8% | 2% | 0% | 42% |
| 75^th^ percentile | 100% | 1% | 27% | 0% | 9% | 2% | 0% | 42% |
| Maximum | 100% | 2% | 30% | 0% | 10% | 2% | 0% | 36% |

Supplementary Table S3: Abatement costs projections (as % of GDP) for the world and six major emitters to net-zero targets for 2050 and IMAGE current policies from the full-factorial analysis.

|  | World | Brazil | China | EU-27 | India | Indo-nesia | USA |
| --- | --- | --- | --- | --- | --- | --- | --- |
| Number of feasible scenarios | 574 | 477 | 648 | 534 | 648 | 648 | 648 |
| Mean | -1.1 | -0.7 | -0.4 | -1.0 | -1.0 | -1.0 | -0.8 |
| Standard deviation | 0.3 | 1.2 | 0.3 | 0.2 | 0.4 | 0.3 | 0.2 |
| minimum | -1.4 | -4.3 | -1.2 | -1.2 | -2.7 | -1.6 | -1.8 |
| 25^th^ percentile | -1.2 | -1.0 | -0.4 | -1.1 | -1.3 | -1.2 | -0.8 |
| Median | -1.1 | 0.0 | -0.3 | -1.0 | -1.0 | -1.0 | -0.7 |
| 75^th^ percentile | -1.0 | 0.0 | -0.1 | -0.8 | -0.8 | -0.8 | -0.6 |
| Maximum | -0.7 | 0.0 | 0.0 | -0.6 | -0.3 | -0.4 | -0.5 |

S4. Supplementary Figures


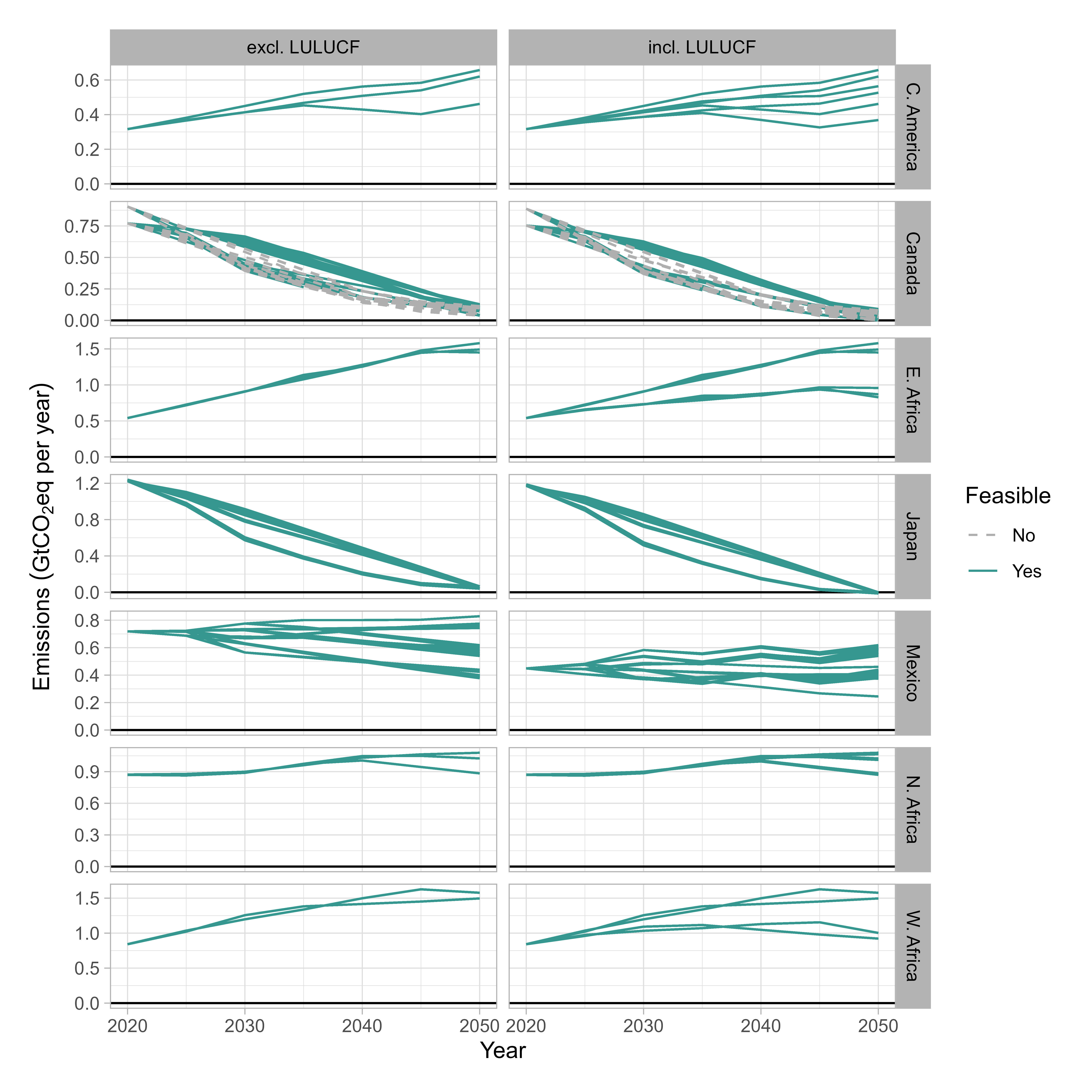


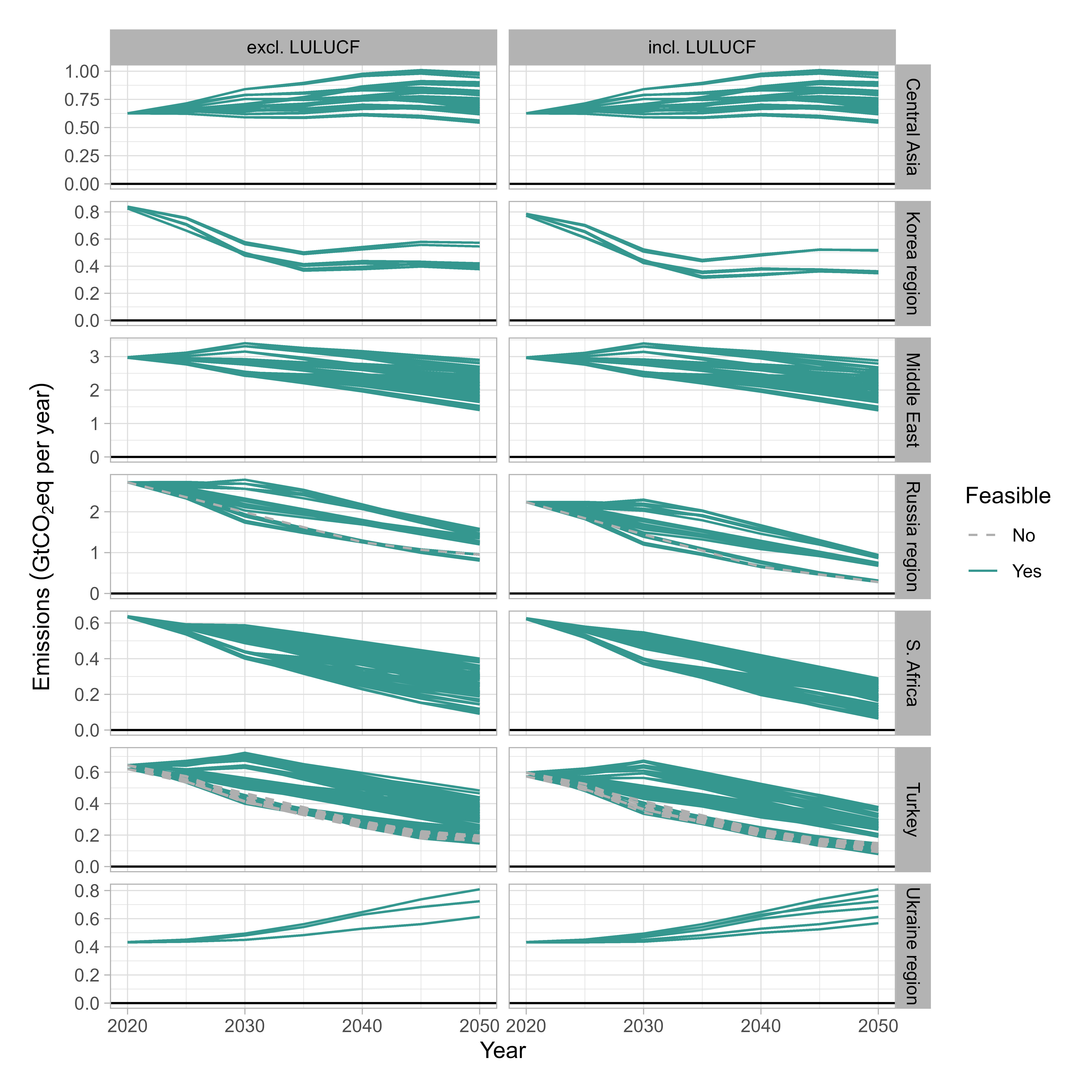

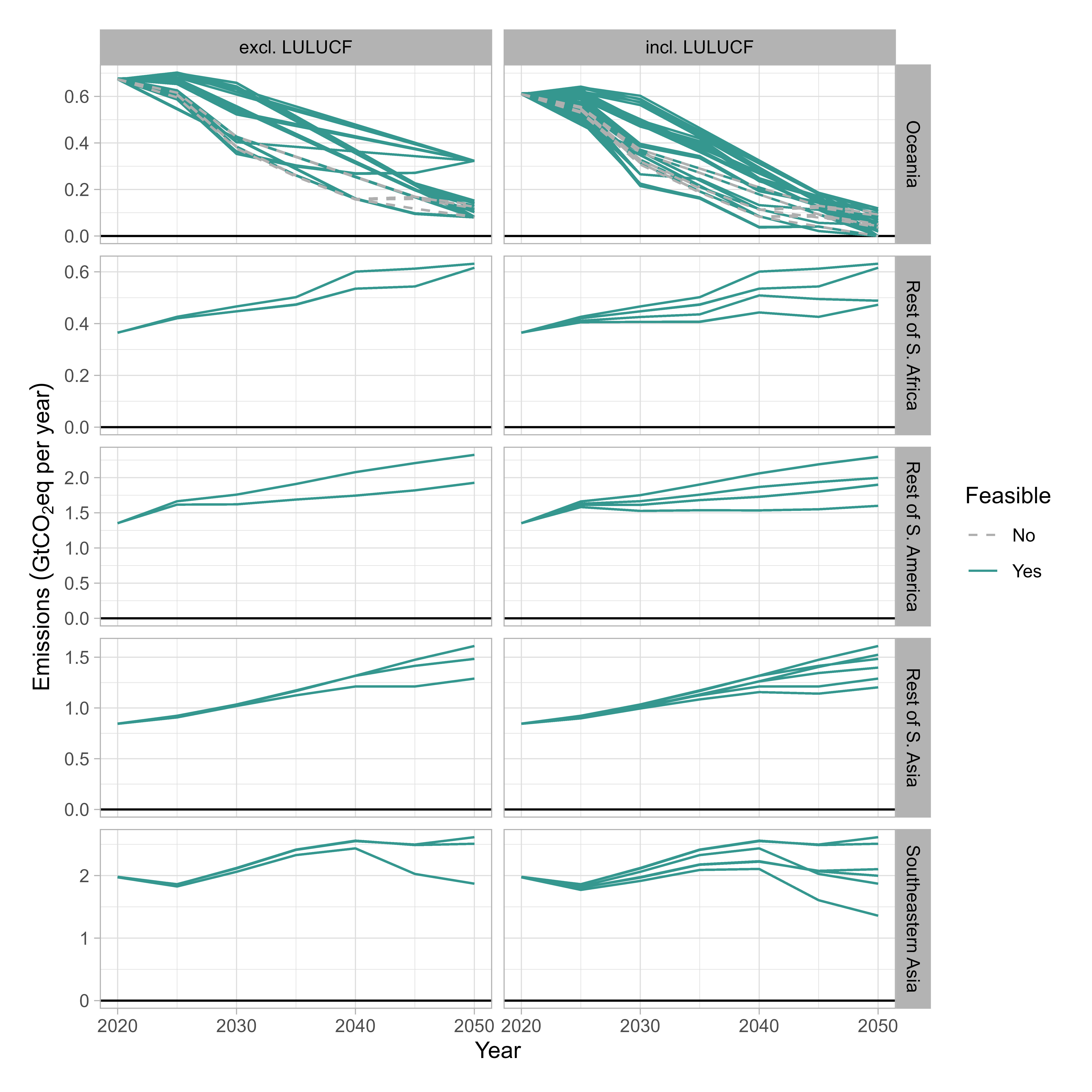


Supplementary Figure S1: Neto-zero emissions corridors for the remaining nineteen regions (showing the full and technically feasible ranges). Figure S3 (main text) gives the corridors for the six major emitting regions.

References

1. Stehfest, E., D.P. Van Vuuren, T. Kram*, et al.* 2014. *Integrated assessment of global environmental change with IMAGE 3.0. Model description and policy applications*. PBL Netherlands Environmental Assessment Agency. The Hague, the Netherlands, <https://www.pbl.nl/en/publications/integrated-assessment-of-global-environmental-change-with-IMAGE-3.0>.

2. Nascimento, L., T. Kuramochi, G. Iacobuta*, et al.* 2022. Twenty years of climate policy: G20 coverage and gaps. *Climate Policy*. **22**: 158-174. <https://doi.org/10.1080/14693062.2021.1993776>.

3. Nascimento, L., M. den Elzen, T. Kuramochi*, et al.* 2024. Comparing the Sequence of Climate Change Mitigation Targets and Policies in Major Emitting Economies. *Journal of Comparative Policy Analysis: Research and Practice*. **26**: 233-250. <https://doi.org/10.1080/13876988.2023.2255151>.

4. Nascimento, L., T. Kuramochi, S. Woollands*, et al.* Greenhouse gas mitigation scenarios for major emitting countries. Analysis of current climate policies and mitigation commitments: 2023 Update. NewClimate Institute (Cologne, Germany), PBL (The Hague, the Netherlands), IIASA (Austria), <https://newclimate.org/resources/publications/emissions-scenarios-for-major-economies-2023-update>.

5. Roelfsema, M., H.L. van Soest, M. den Elzen*, et al.* 2022. Developing scenarios in the context of the Paris Agreement and application in the integrated assessment model IMAGE: a framework for bridging the policy-modelling divide. *Environmental Science and Policy* **135**: 104-116. <https://doi.org/10.1038/s41467-020-15414-6>.

6. Roelfsema, M., H.L. van Soest, M. Harmsen*, et al.* 2020. Taking stock of national climate policies to evaluate implementation of the Paris Agreement. *Nature Communications*. **11**: 2096. <https://doi.org/10.1038/s41467-020-15414-6>.

7. Dafnomilis, I., H.-H. Chen, M. den Elzen*, et al.* 2022. Targeted Green Recovery Measures in a Post-COVID-19 World Enable the Energy Transition. *Frontiers in Climate*. **4**. <https://www.frontiersin.org/articles/10.3389/fclim.2022.840933>.

8. van Soest, H.L., L. Aleluia Reis, L.B. Baptista*, et al.* 2021. Global roll-out of comprehensive policy measures may aid in bridging emissions gap. *Nature Communications*. **12**: 6419. <https://doi.org/10.1038/s41467-021-26595-z>.

9. Hof, A.F., K. Esmeijer, H.S. de Boer*, et al.* 2022. Regional energy diversity and sovereignty in different 2 °C and 1.5 °C pathways. *Energy*. **239**: 122197. <https://www.sciencedirect.com/science/article/pii/S0360544221024452>.

10. Stehfest, E., D.P. van Vuuren, A.F. Bouwman*, et al.* 2014. *Integrated Assessment of Global Environmental Change with IMAGE 3.0. Model description and policy applications*. the Hague: PBL Netherlands Environmental Assessment Agency, <http://www.pbl.nl/en/publications/integrated-assessment-of-global-environmental-change-with-IMAGE-3.0>.

11. van Vuuren, D.P., E. Stehfest, D. Gernaat*, et al.* 2017. Energy, land-use and greenhouse gas emissions trajectories under a green growth paradigm. *Global Environmental Change*. **42**: 237–250. <http://www.sciencedirect.com/science/article/pii/S0301421515302081>.

12. van Vuuren, D.P., E. Stehfest, D.E.H.J. Gernaat*, et al.* 2018. Alternative pathways to the 1.5 °C target reduce the need for negative emission technologies. *Nature Climate Change*. **8**: 391-397. <https://doi.org/10.1038/s41558-018-0119-8>.

13. den Elzen, M.G.J., A. Hof, M. van den Berg*, et al.* 2014. "Climate policy". In *Integrated Assessment of Global Environmental Change with IMAGE 3.0 - Model description and policy applications*. E. Stehfest, D. Van Vuuren, T. Kram, et al., Eds.: 71-152. The Hague: PBL.

14. Hof, A.F., M.G.J. den Elzen, A. Admiraal*, et al.* 2017. Global and regional abatement costs of Nationally Determined Contributions (NDCs) and of enhanced action to levels well below 2 °C and 1.5 °C. *Environmental Science & Policy*. **71**: 30-40. <https://doi.org/10.1016/j.envsci.2017.02.008>.

15. Harmsen, J.H.M., D.P. van Vuuren, D.R. Nayak*, et al.* 2019. Long-term marginal abatement cost curves of non-CO2 greenhouse gases. *Environmental Science & Policy*. **99**: 136-149. <https://doi.org/10.1016/j.envsci.2019.05.013>.

16. Harmsen, M., C. Tabak, L. Höglund-Isaksson*, et al.* 2023. Uncertainty in non-CO2 greenhouse gas mitigation contributes to ambiguity in global climate policy feasibility. *Nature Communications*. **14**: 2949. <https://doi.org/10.1038/s41467-023-38577-4>.

17. EPA. (United States Environmental Protection Agency (EPA), Washington DC, report EPA-430-R-13-011, <http://www.epa.gov/climatechange/Downloads/EPAactivities/MAC_Report_2013.pdf>). Global Mitigation of Non-CO2 Greenhouse Gases: 2010-2030.

18. van Vuuren, D.P., M.G.J. den Elzen, B. Eickhout*, et al.* 2007. Stabilizing greenhouse gas concentrations at low levels: an assessment of reduction strategies and costs. *Climatic Change*. **81**: 119-159. <https://doi.org/10.1007/s10584-006-9172-9>.

19. Hoogwijk, M., A. Faaij, B. de Vries*, et al.* 2009. Exploration of regional and global cost–supply curves of biomass energy from short-rotation crops at abandoned cropland and rest land under four IPCC SRES land-use scenarios. *Biomass and Bioenergy*. **33**: 26-43. <https://doi.org/10.1016/j.biombioe.2008.04.005>.

20. Meinshausen, M., S.C.B. Raper & T.M.L. Wigley. 2011. Emulating coupled atmosphere-ocean and carbon cycle models with a simpler model, MAGICC6 - Part 1: Model description and calibration. *Atmospheric Chemistry and Physics*. **11**: 1417-1456. <https://doi.org/10.5194/acp-11-1457-2011>.

21. Hauglustaine, D.A., Y. Balkanski & M. Schulz. 2014. A global model simulation of present and future nitrate aerosols and their direct radiative forcing of climate. *Atmospheric Chemistry and Physics*. **14**: 11031-11063. <https://doi.org/10.5194/acp-14-11031-201>.

22. Meinshausen, M., S.J. Smith, K. Calvin*, et al.* 2011. The RCP greenhouse gas concentrations and their extensions from 1765 to 2300. *Climatic Change*. **109**: 213-241. <https://doi.org/10.1007/s10584-011-0156-z>.

23. Forsell, N., Z. Araujo Gutiérrez & M. Chen. 2024. Historical and future development of greenhouse gas emission and removal from the land use sector from the view of countries. *FORMATH*. **23**. <https://doi.org/10.15684/formath.23.003>.

24. Friedlingstein, P., M.W. Jones, M. O'Sullivan*, et al.* 2022. Global carbon budget 2021. *Earth System Science Data*. **14**: 1917-2005, <https://doi.org/1910.5194/essd-1914-1917-2022>.

25. Grassi, G., J. House, W.A. Kurz*, et al.* 2018. Reconciling global-model estimates and country reporting of anthropogenic forest CO2 sinks. **8**: 914-920. <https://doi.org/10.1038/s41558-018-0283-x>.

26. Grassi, G., G. Conchedda, S. Federici*, et al.* 2022. Carbon fluxes from land 2000–2020: bringing clarity to countries' reporting. *Earth System Science Data*. **14**: 4643-4666. <https://doi.org/10.5194/essd-14-4643-2022>.

27. Grassi, G., J. House, F. Dentener*, et al.* 2017. The key role of forests in meeting climate targets requires science for credible mitigation. *Nature Clim. Change*. **7**: 220-226. <http://dx.doi.org/10.1038/nclimate3227>.

1. www.climatepolicydatabase.org [↑](#footnote-ref-2)
